# Knockdown of PSCA induces EMT and decreases metastatic potentials of the human prostate cancer DU145 cells

**DOI:** 10.1186/s12935-016-0295-4

**Published:** 2016-03-15

**Authors:** Ran Kang, Shankun Zhao, Luhao Liu, Futian Li, Ermao Li, Lianmin Luo, Lihua Xu, ShawPong Wan, Zhigang Zhao

**Affiliations:** Department of Urology & Andrology, Minimally Invasive Surgery Center, The First Affiliated Hospital of Guangzhou Medical University, Guangdong Provincial Key Laboratory of Urology, No.1-3, Kangda Road, Guangzhou, 510230 Guangdong Province China; Department of Hematology, The First Affiliated Hospital of Guangzhou Medical University, Guangzhou, 510230 China

**Keywords:** Prostate cancer, Prostate stem cell antigen, Epithelial-mesenchymal transition, Metastasis

## Abstract

**Background:**

Prostate stem cell antigen (PSCA) expression has been shown to correlate with prostatic carcinogenesis and prostate cancer (PCa) progression. The underlying mechanisms for these processes are currently unknown. Epithelial to mesenchymal transition (EMT) has been associated with the invasiveness and the distant metastasis of PCa. In this study, we investigated the effects of knocking down the PSCA on the cell migration, the invasiveness, and the EMT of the PCa cell line DU145 in vitro and in vivo.

**Methods:**

Four target sequences of the small hairpin RNA for PSCA were designed, and the best effect knockdown sequence shRNA#1 was screened to construct the stable transfected DU145 cell line (DU145 shRNA#1), the scramble sequence was also designed to construct the stable transfected DU145 cell line(DU145 scramble). Cell migration and invasion were studied using Transwell assay. Quantitative RT-PCR, Western blot (WB) were used to quantify PSCA, E-cadherin, β-catenin, Vimentin, Fibronectin expression in DU145, DU145 scramble, DU145 shRNA#1 in vitro and in vivo. RT-PCR, immunofluorescent staining were used to quantify PSCA, E-cadherin, and Vimentin expression in vitro. EMT-related genes Snail, Slug, and Twist, were quantified by quantitative RT-PCR in vitro.

**Results:**

The constructed stable knockdown of the PSCA in the DU145 cell had a silencing effect up to 90.5 %. DU145 shRNA#1 became scattered from the tightly packed colonies. It was associated with decreased cell migration and invasion. There was also an increased Vimentin and Fibronectin expression, an inhibited E-cadherin and β-catenin expression at both the mRNA and the protein levels when compared to the DU145 and the DU145 scramble in vitro and vivo. Furthermore, with the exception of the Snail, the expression of EMT-related Slug and Twist genes were upregulated.

**Conclusions:**

Our data indicated that knockdown of PSCA induced EMT and reduced metastatic potentials of the DU145 cells, suggesting that PSCA played an important role in prostatic carcinogenesis and progression.

## Background

Prostate cancer is the most commonly diagnosed cancer and the second leading cause of cancer-related death in men in the Western countries. According to the newest statistics, an estimated 220,800 cases of prostate cancer in the United States will be diagnosed. It will account for 26 % of all newly diagnosed cancers and will result in an estimated 27,540 deaths in 2015 [1].

There remain significant challenges in the management of this disease, especially since many of the patients do eventually progress to metastatic disease. Androgen ablation is the first line treatment for the metastatic prostate cancer. However most of the patients will eventually become castration-resistant [2]. Currently there is no effective treatment for the metastatic disease. Gene therapy seems to be a potential promising modality. It has been reported by many researchers and the effectiveness has been validated [3, 4]. Guo et al. [5] have postulated the possibility of developing a RNAi-based therapy for the prostate cancer. To do so a suitable treatment target would be needed.

Reiter et al. [6] evaluated many antigens in the LAPC-4 xenograft model using representational difference analysis (RDA). They confirmed that the prostate stem cell antigen (PSCA) expression was prostate specific and it was overexpressed in the majority of the prostate cancers. PSCA is located on the chromosome 8q24.2. It is composed of 660 bp and possesses an amino-terminal signal sequence, a carboxyl-terminal GPI anchoring sequence, and numerous N-glycosylation sites. Subsequently, many researchers have proven the relationship between PSCA and prostate cancer. Based on Reiter’s research, Gu et al. [7] found that as the tumor stage and the Gleason scores increased so was the PSCA expression. It was especially noticeable in the cases of bony metastases. Following Gu’s research, Han et al. [8] showed that Gleason score, tumor stage, seminal vesicle invasion, capsular penetration, as well as the biochemical recurrence were all closely correlated to the increased PSCA expression. Lam et al. [9] detected PSCA mRNA and protein expression in the clinical specimens of prostate cancer. They showed that prostate cancer metastases, especially the bony metastases, had increased PSCA expression. Our group also reported that PSCA expression positively correlated with the increased pathological grade (poor cell differentiation), heightened clinical stage, and androgen-independence. We speculated PSCA might even have a role in the prostate carcinogenesis [10–12]. There was increasing evidence to show that PSCA was a favorable antigen for the diagnosis and treatment of prostate cancer, especially for the gene therapy.

Tumor metastasis is a complicate process. The cancer cell has first to acquire the ability to invade into the adjacent organs and/or to migrate through bloodstream or lymphatic system to distant sites and to survive [13]. The same is true for the prostate cancer metastasis, especially since most of the prostate cancer metastasis occur in the lymph nodes and/or in the bones. The role of PSCA in the prostate cancer cell migration and invasion has yet been elucidated.

Epithelial to mesenchymal transition (EMT) in PCa was first proposed by Greenburg et al. [14]. They found epithelial cells could transform into mesenchymal cells in the collagen gels. Subsequently, research on the EMT had gained broad interests. Currently, EMT was characterized by a decrease of the epithelial markers such as E-cadherin, an increase of the mesenchymal markers such as Vimentin, loss of polarity, lower adhesiveness, and higher mobility. EMT was considered to play a critical role in the tumor metastasis [15, 16]. There was ample evidence that EMT-like states occurred in and might contribute to the PCa metastasis. Therefore, it has become a very active area of research [17]. Ding et al. and Gao et al. demonstrated that when EMT occurred in PCa cells, the cells would acquire mobility and invasive potential [18, 19] .

In order to elucidate the precise function of PSCA in the PCa and the mechanism of PSCA in the PCa metastasis, we constructed a target sequence of the small hairpin RNA for the PSCA. Retrovirus was then used as a vector to create a stable transfected PCa DU145 cell line. We next studied the cell migration and invasion, as well as the epithelial-to-mesenchymal transition with the knockdown of PSCA in the DU145 cells.

## Results

### PSCA was highly expressed in the metastatic DU145 prostate cancer cells

**Fig. 1:**
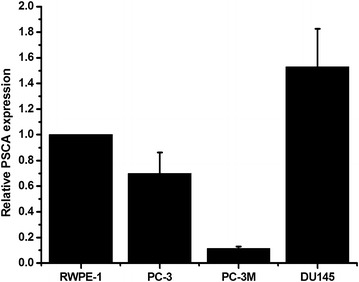
PSCA is highly expressed in the metastatic DU145 prostate cancer cells. The expression of PSCA in various prostate cells was analyzed by qRT-PCR, GAPDH was used as a loading control

To investigate the role of PSCA in the PCa metastatic potentials, the levels of PSCA mRNA in various prostate cell lines were assessed. It was found that the PSCA was highly expressed in the DU145 metastatic PCa cells. In this study, DU145 cells with relative higher PSCA expression were selected to perform the experiments (Fig. 1).

### Successful construction of stable knockdown of PSCA DU145 cell lines

**Fig. 2:**
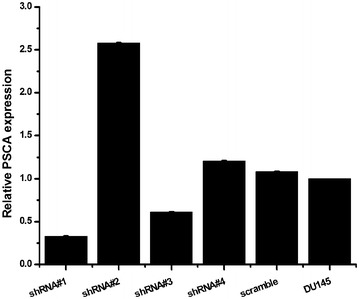
Screened the silencing effect of the target sequence of the small hairpin RNA. Designed four target sequences of the small hairpin RNA for the PSCA, and one scrambled control, and what we screened the best silencing sequence was PSCA shRNA#1

**Fig. 3:**
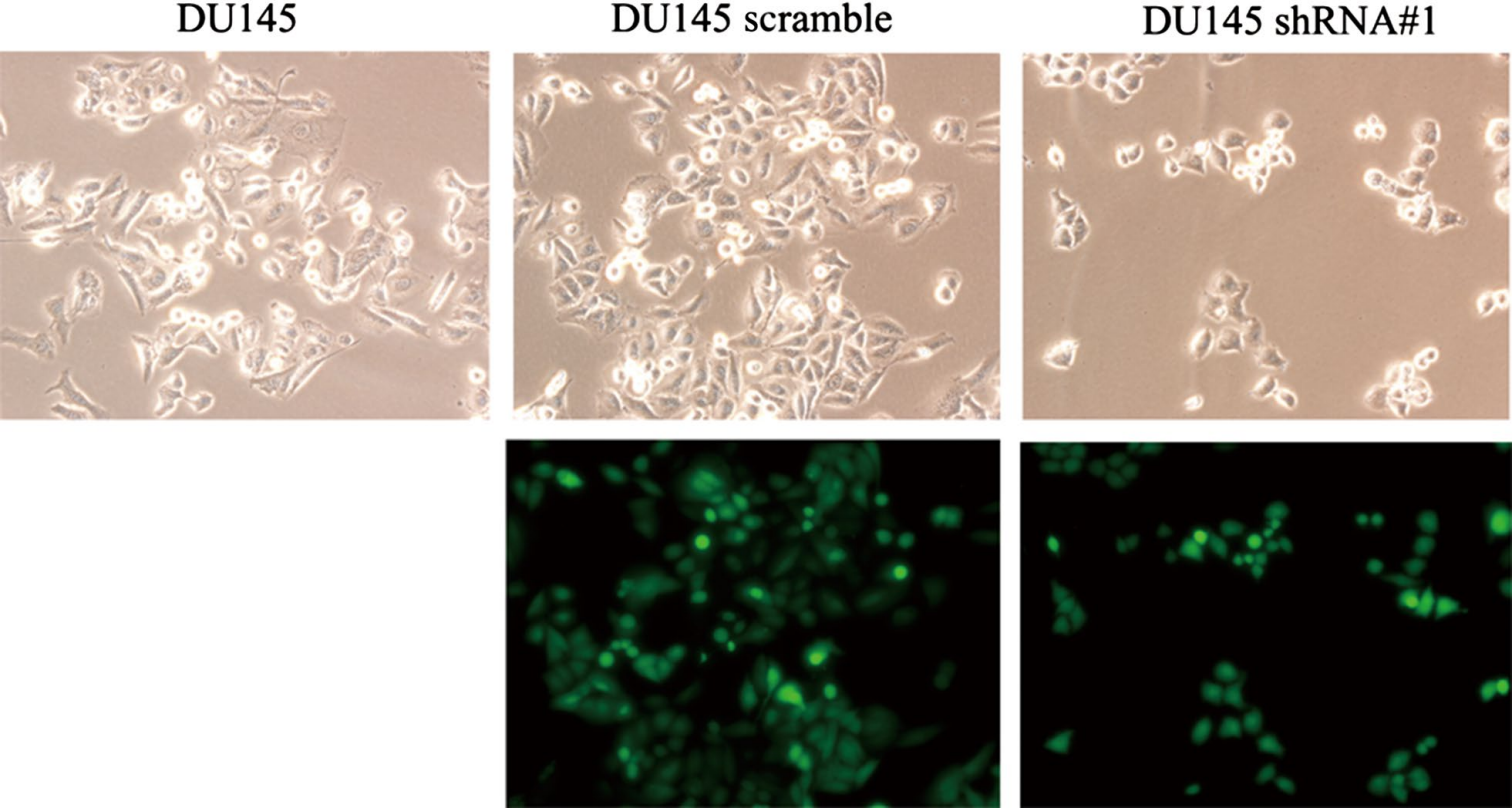
The cell lines photographs of DU145, DU145 scramble, DU145 shRNA#1 DU145 *white light* picture (×200), DU145 scramble *white light* picture and *Fluorescent* images (×200), DU145 shRNA#1 *white light* picture and *Fluorescent* images (×200)

**Fig. 4:**
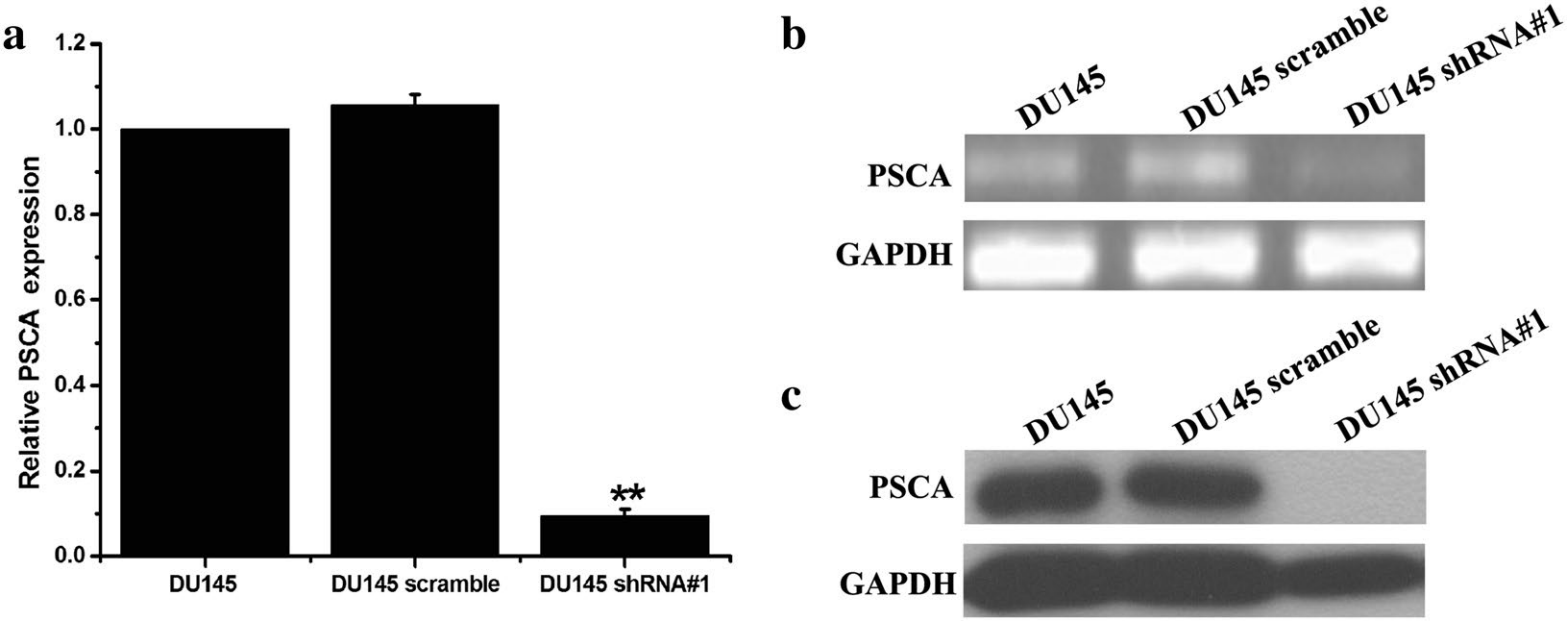
Successful construction of stable knockdown of PSCA DU145 cell lines. **a** The mRNA level of PSCA was analyzed by QRT-PCR among DU145, DU145 scramble and DU145 shRNA#1. QRT-PCR indicated that the silencing PSCA level was up to 90.5 %. **b** The mRNA level of PSCA was analyzed by RT-PCR. **c** The protein level of PSCA was analyzed by Western blot **P < 0.001, show DU145VS DU145 shRNA#1

**Fig. 5:**
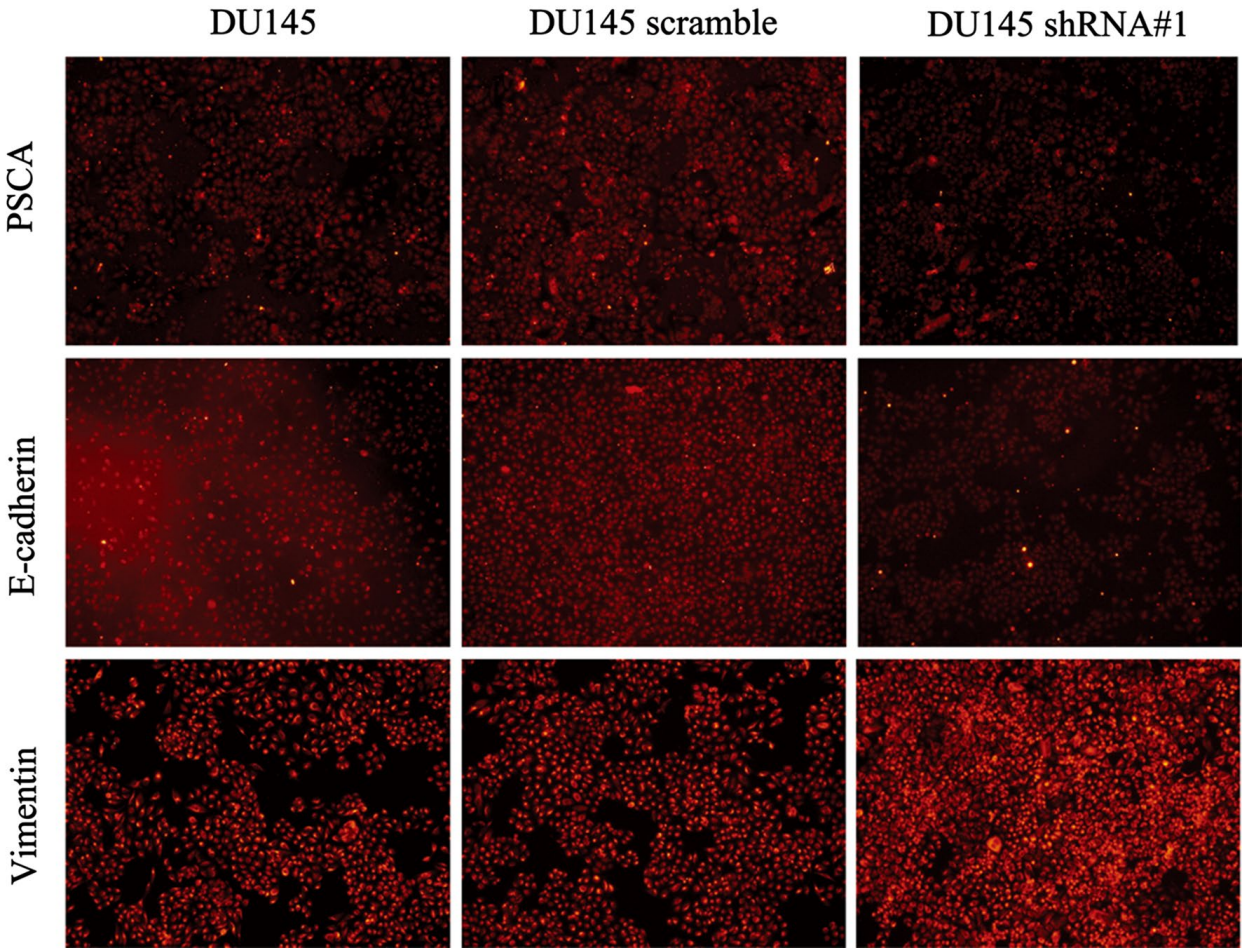
Immunofluorescent staining. PSCA, E-cadherin, Vimentin was analyze by immunofluorescent staining. The *results* indicated that knock down of PSCA can decreased the expression of E-cadherin, and elevated the expression of Vimentin

The target sequence of the small hairpin RNA for the PSCA was designed. The sequence was then annealed and cloned into the psi-LVRH1GP vector. The psi-LVRH1GP-shRNA was sequenced. The results indicated that the construction of the recombined plasmid was successful. The best effect sequence of knockdown was screened, shRNA#1 was the best target knockdown sequence (Fig. 2). In the study, the vectors were co-transfected along with the Gag/Pol, Rev, VSV-G vectors into the 293T cells. The retroviruses were added into DU145 cells for the infection. The positive clones were selected in puromycin (5 ug/ml). The stable PSCA transfectants were isolated after 2 weeks of selection. The photographs of the stable knockdown of PSCA cell lines were shown in Fig. 3. QRT-PCR indicated that the silencing effect was up to 90.5 % (Fig. 4a). RT-PCR (Fig. 4b), WB (Fig. 4c), and immunofluorescent staining (Fig. 5) were also used to determine the knockdown level.

### Knockdown of PSCA reduced cell migration and invasion in prostate cancer DU145 cells

**Fig. 6:**
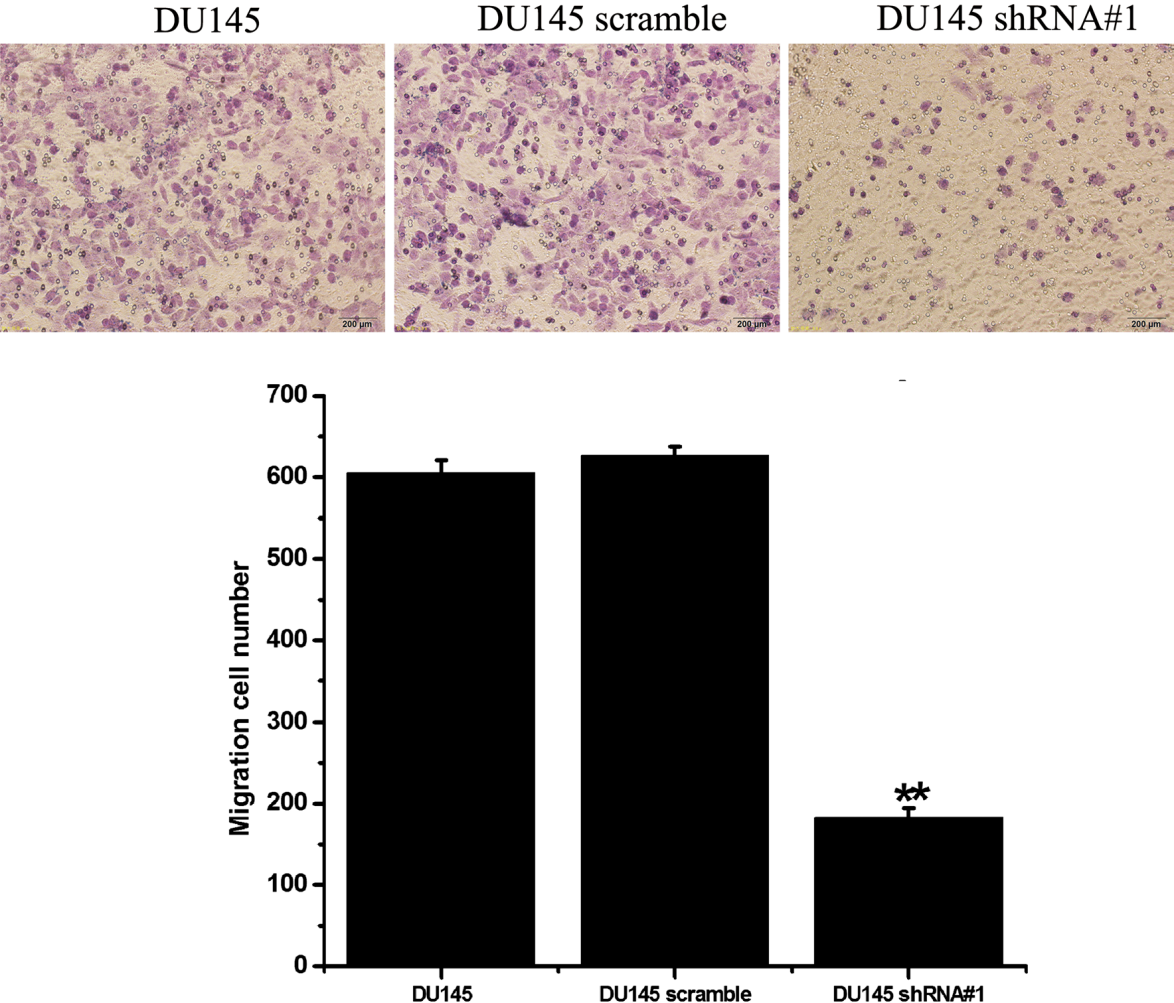
Knockdown of PSCA decreased cell migration of DU145 cells, which were assessed by a transwell-based motility assay after migration 24 h (original magnifications: ×200). **P < 0.001, show DU145 VS DU145 shRNA#1

**Fig. 7:**
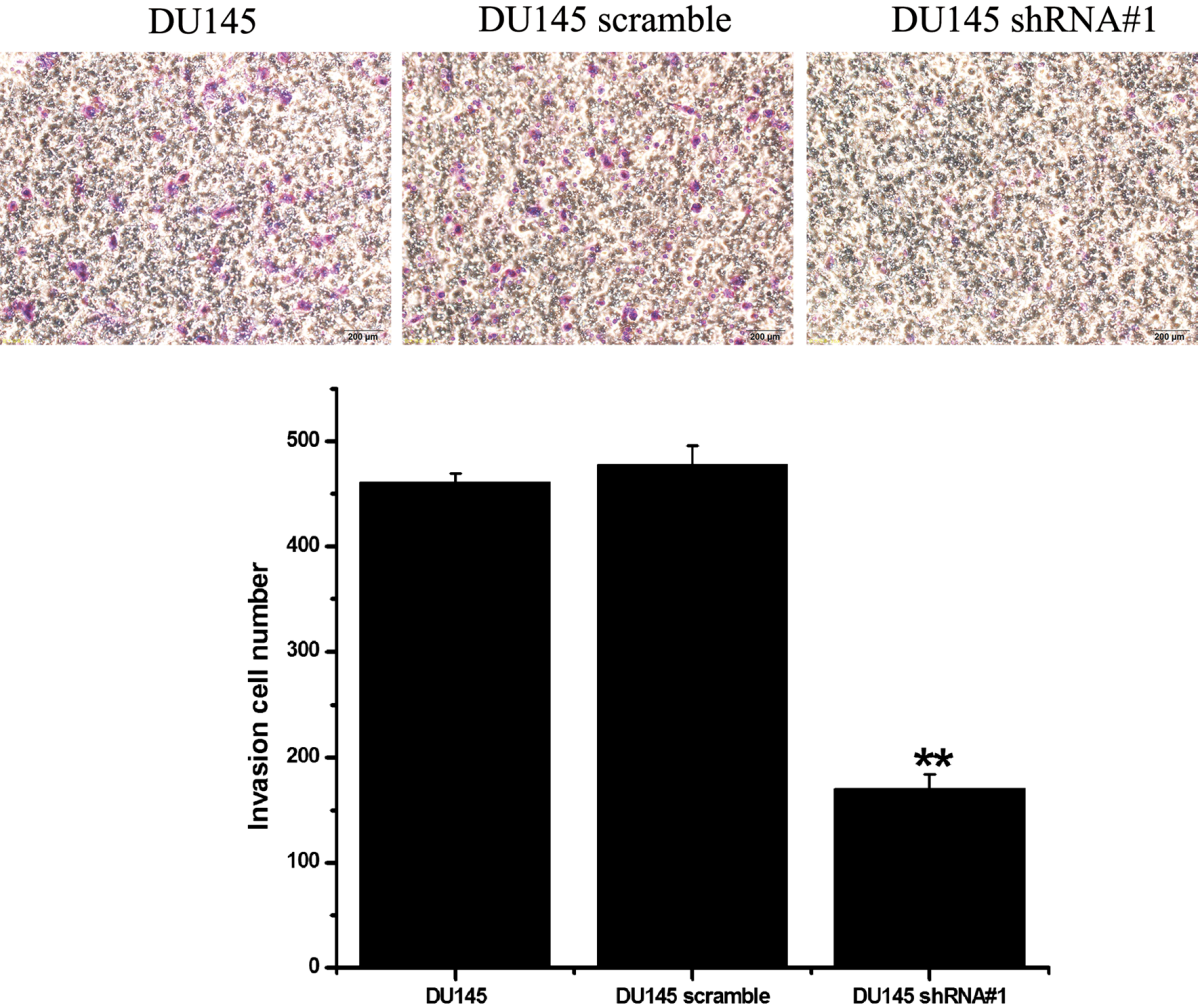
Knockdown of PSCA decreased cell invasion of DU145 cells knockdown of PSCA decreased cell invasion of DU145 cells, which were assessed by a transwell-based invasion assay after 24 h (original magnifications: ×200). **P < 0.001, show DU145 VS DU145 shRNA#1

The effects of knockdown of PSCA on the cell motility of the PCa cell lines were assessed using the Transwell migration assay. To minimize the impact of the differential cell growth rates on the motility assay, we induced cell cycle arrest by maintaining cells under serum-starvation conditions for 24 h before performing the motility assay. As shown in Fig. 6, knockdown of PSCA significantly reduced the cell migration of the DU145 cells as compared to the control DU145 (P < 0.001) and to the DU145 scramble (P < 0.001). Knockdown of PSCA expression also remarkably reduced the number of invasive cells as compared to the control group (P < 0.001, for all) (Fig. 7). These consistent results suggested that abolition of the expression of PSCA could effectively reduce the metastatic potentials of the DU145.

### Knockdown of PSCA induced epithelial-to-mesenchymal transition in vitro

**Fig. 8:**
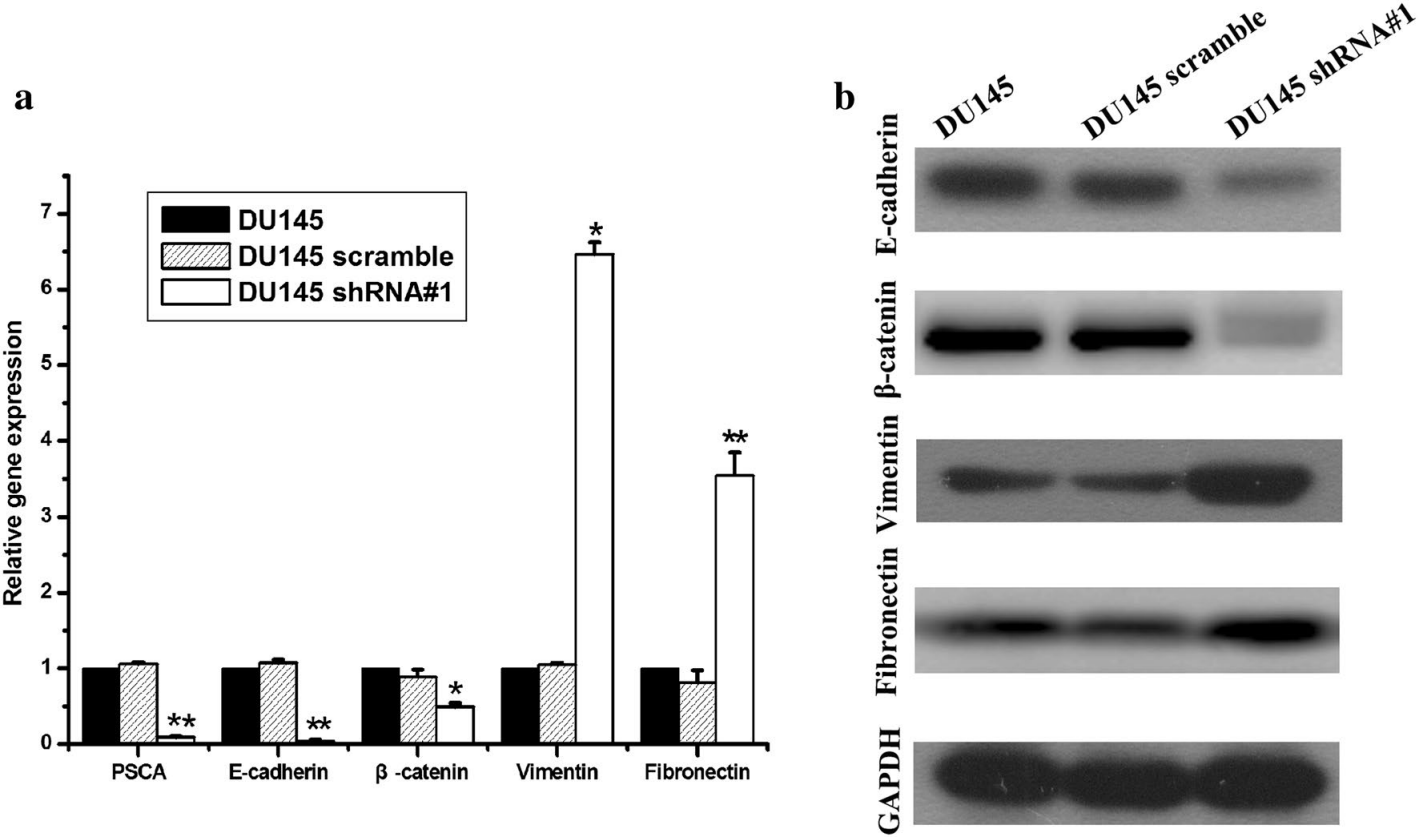
Knock down of PSCA regulated the expression of EMT markers in DU145 cells. **a** knock down of PSCA lead to downregulation of the E-cadherin, β-catenin and upregulation of the Vimentin, Fibronectin expression. The expression of E-cadherin, β-catenin, Vimentin, and Fibronectin was detected by qRT-PCR. GAPDH was used as loading control. **P < 0.001, show DU145 VS DU145 shRNA#1. *P < 0.01, show DU145 VS DU145 shRNA#1. **b** knock down of PSCA lead to downregulation of the E-cadherin, β-catenin, and upregulation of the Vimentin, Fibronectin expression. The expression of E-cadherin, β-catenin, Vimentin, Fibronectin was detected by Western Blot

As shown in Fig. 3, DU145 shRNA#1 cells became scattered from the tightly packed colonies when compared to the parental DU145 and the DU145 scramble cells. This represented one of the characteristics of EMT. To further determine whether PSCA induced the molecular alterations of EMT in these cells, the expressions of the epithelial markers E-cadherin, β-catenin and the mesenchymal marker Vimentin, Fibronectin were examined. QRT-PCR showed that the knockdown of PSCA lead to downregulation of the E-cadherin, β-catenin and upregulation of the Vimentin, Fibronectin expression (Fig. 8a). The results from Western blot were consistent with those from qRT-PCR (Fig. 8b). Immunofluorescent staining of E-cadherin and Vimentin showed the same results as above (Fig. 5).

### Knockdown of PSCA induced EMT relating genes in DU145

**Fig. 9:**
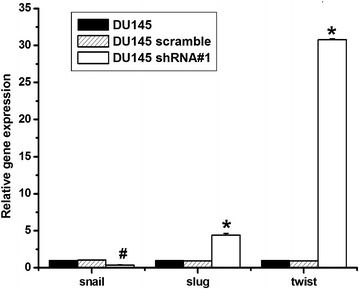
Knockdown of PSCA induced EMT relating genes in DU145 Knock down of PSCA increased slug and twist expression, but decreased snail expression by qRT-PCR. *P < 0.01, show DU145 VS DU145 shRNA#1. ^#^P < 0.05, show DU145 VS DU145 shRNA#1

One of the hallmarks of EMT is the loss of E-cadherin. E-cadherin transcription is silenced in many tumor and is considered to be a tumor suppressor. The zinc-finger containing the Snail and Slug proteins and the helix-loop-helix transcription factor Twist can suppress the E-cadherin expression and induce EMT. To check whether these transcription factors were regulated by PSCA, the transcriptional levels of E-cadherin regulators in the DU145 cells were assessed. The expression of the Slug and the Twist was increased but the Snail was downregulated in the EMT-like DU145 shRNA#1 cells (Fig. 9). This suggested that the Snail might have distinct functions in the DU145 cells. These results indicated that PSCA regulated the EMT promoting genes such as the Slug and Twist, but not the Snail in the DU145 cells. Taken all together, these results supported the idea that the knockdown of PSCA decreased the migratory and invasive behavior and induced EMT in DU145 in vitro.

### Knockdown of PSCA induced epithelial-to-mesenchymal transition in vivo

**Fig. 10:**
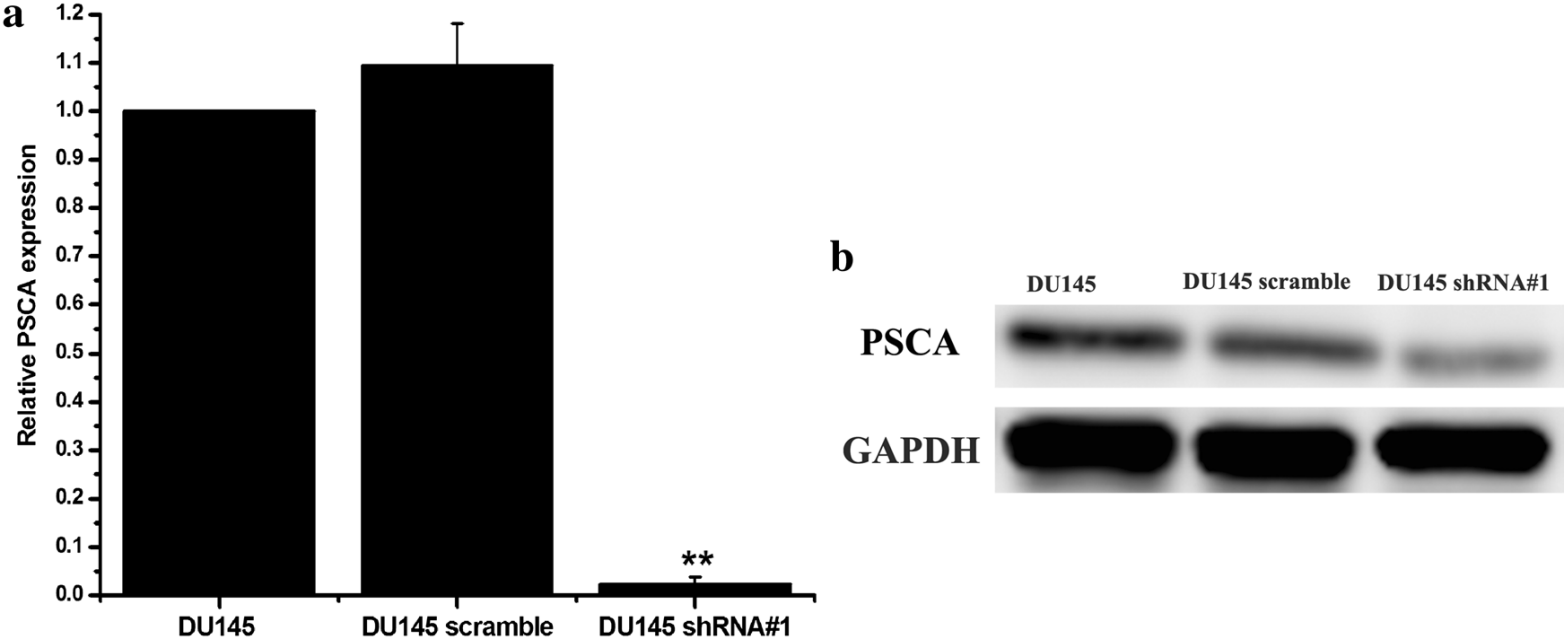
PSCA expression level in xenograft tumor. **a** The mRNA level of PSCA was analyzed by qRT-PCR in xenograft tumor. **b** The protein level of PSCA was analyzed by Western blot in xenograft tumor. **P < 0.001, show DU145 VS DU145 shRNA#1

To further explore whether knockdown of PSCA induced epithelial-to-mesenchymal transition in vivo, we conducted xenograft tumor model assays by subcutaneously injecting DU145, DU145 scramble, and DU145 shRNA#1 cells into the right side of axillary of mice. The subcutaneous tumors were excised. QRT-PCR was conducted to confirm that DU145 shRNA#1 had the lower PSCA level than DU145, DU145 scramble (P < 0.001, for all) (Fig. 10a). In consistent with the mRNA level, Western blot had the same results (Fig. 10b). Our results suggested that PSCA in DU145 shRNA#1 xenograft tumor had also the lowest level than control (P < 0.001, for all).

**Fig. 11:**
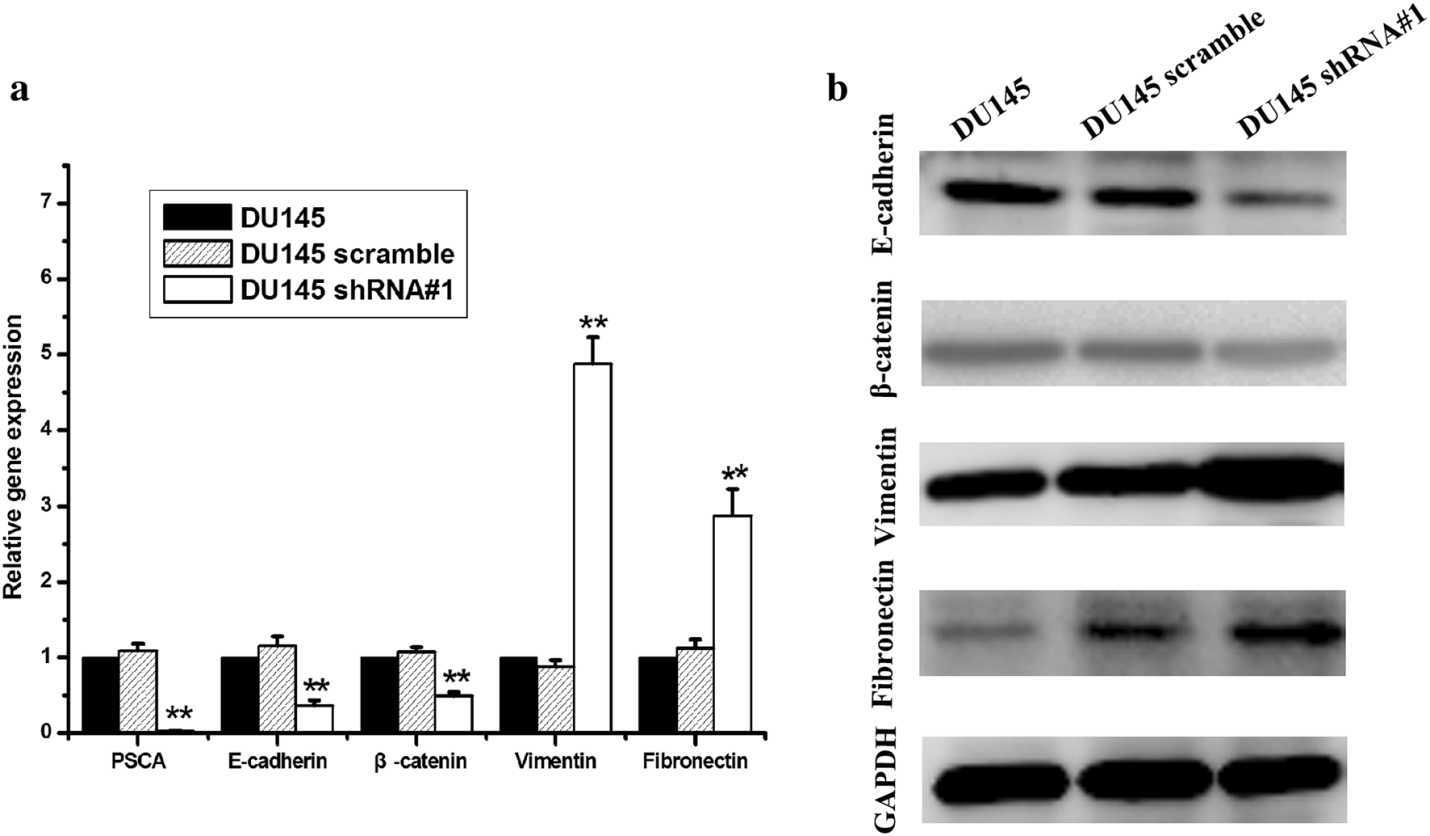
Knock down of PSCA regulated the expression of EMT markers in DU145 xenograft tumor.** a** QRT-PCR was conducted to confirm the mRNA level of E-cadherin, β-catenin, Vimentin, and Fibronectin in DU145, DU145 scramble, DU145 shRNA#1xenograft tumor.** b** The protein level of E-cadherin, β-catenin, Vimentin, and Fibronectin was analyzed by Western blot, GAPDH was used as loading control. **P < 0.001, show DU145 VS DU145 shRNA#1

In order to explore if EMT occured in vivo, we detected markers of EMT of E-cadherin,β-catenin, Vimentin, Fibronectin. QRT-PCR was conducted to confirm that DU145 shRNA#1 had the lower level of E-cadherin, β-catenin than DU145, DU145 scramble (P < 0.001, for all), and also had the higher level of Vimentin, Fibronectin than DU145, DU145 scramble (Fig. 11a, P < 0.001, for all). In consistent with the mRNA level, Western blot indicated the same results. (Figure 11b), which indicated EMT also occured in vivo.

## Discussion

In this study, we designed four target sequences of a small hairpin RNA for PSCA and screened the best effect sequence for a knockdown. We then constructed the stable transfected DU145 cells. The silencing effect of the DU145 shRNA#1 attained to 90.5 % when compared to the control DU145. These data indicated that the experimental knockdown of PSCA had met our objective. We found that when knockdown of PSCA, DU145 shRNA#1 migration to the bottom of the Transwell significantly decreased when compared to the DU145 and the DU145 scramble. In addition, DU145 shRNA#1 also demonstrated significantly reduced invasiveness through the BD matrigel as compared to the DU145 and the DU145 scramble. These results indicated that knockdown of PSCA decreased the metastatic potentials of the PCa DU145 cells in vitro. Our results was in agreement with our prior study [20], in which we reported that silencing the PSCA using siRNA could inhibit the proliferative and the invasive properties of the human PCa cells PC3M. Combining with our prior findings, the conclusion that the knockdown of PSCA decreased the metastatic potentials of prostate cancer cells in vitro was reaffirmed.

In order to elucidate the mechanism affecting the metastatic potentials of the DU145, we investigated the EMT with the knock down of the expression of PSCA in the DU145 cell in vitro and vivo. In this study, we observed the morphologic change of DU145 shRNA#1 cells. This was characterized by the transformation from tightly packed colonies to scattered phenotype when compared to DU145 and DU145 scramble cells. Knockdown of PSCA also downregulated the epithelial marker E-cadherin, β-catenin and upregulated the mesenchymal marker Vimentin, Fibronectin at the mRNA and protein level in vitro and in vivo. In addition we found that the EMT-related genes Slug and Twist were elevated, but the Snail was downregulated. Further study is needed to elucidate this distinct function of the Snail in DU145 cell.

EMT was thought to promote prostate cancer metastasis [21–23]. In our study, we observed that the knockdown of PSCA decreased the metastatic potentials and simultaneously induced EMT in DU145. Therefore the decreased metastatic potential was felt to be independently of EMT. Drake et al. [24, 25] isolated the cell line TEM4-18, a subpopulation of PCa PC-3 cell line. They found the TEM4-18 exhibited hallmarks of EMT that were not in the PC-3; however, the TEM4-18 had a decreased migration by the Transwell assay. This was consistent with our findings. In the present study, in vivo results also showed that E-cadherin, β-catenin were downregulated and Vimentin, Fibronectin were upregulated, suggesting an EMT. These results suggested that EMT might not always be associated with the changes of cell-autonomy that were inherent to the invasive behavior, or EMT had a context-dependent role in the metastatic cellular behavior. Further study is needed to confirm these findings in other prostatic cancer cell lines.

PSCA is a glycosylphosphatidylinositol (GPI) anchored cell surface antigen. It shares 30 % homology with the stem cell antigen 2 (SCA-2) and is also homologous to the Thy-1/Ly-6 family. Reiter et al. [6] speculated that PSCA might play an important role in the stem/progenitor cell functions such as self-renewal (anti-apoptosis). Bahrenberg et al. [26] based on his finding that PSCA expression in RT112 cells was dependent on the cell–cell contact and surface adhesion, drew the conclusion that PSCA might be related to the adhesion of the cells. Many authors identified that PSCA might play a role in the cell growth, migration, invasion in vitro, as well as the tumor growth and metastasis in vivo. However, PSCA seemed to have diverse functions in different tumors. Ono et al. [27] observed PSCA could reduce cell-proliferation and inhibit the invasiveness of gallbladder cancer cell lines. It is possible that the PSCA was down-regulated in the gallbladder tumor since the PSCA was a tumor suppressor gene in gallbladder cancer. The specific mechanism was unclear. It deserves to be studied further.

The precise mechanisms of the PSCA regulating the cellular activities in cells are unknown. Marra et al. [28] identified that the knockdown of PSCA delayed bladder cancer cell growth through activating the genes downstream of the IFNα/β receptor. Since the PSCA is a GPI-anchor proteins, Saeki et al. [29] speculated that PSCA might work through two mechanisms. The first possibility was that PSCA might form compounds with another yet unidentified protein that controlled both the transmembrane and the intracellular regions. When PSCA bound to this protein, the resulting complex could activate the downstream signaling molecules. The second possible mechanism was that the phospholipase C decomposed the GPI anchor of the PSCA. The PSCA would then be excreted from the membrane and bound with an appropriate ligand. This would start the next signaling cascades. How the knockdown of PSCA in DU145 cell regulated the cell activities in this study needs to be further elucidated.

A limitation of our study is that only one cell line was studied and the experiments were not performed using additional prostatic cell lines to validate the results. Despite this limitation, the findings presented here demonstrated that knockdown of PSCA could decrease the metastasis and induce the EMT in DU145 cells. The decreased metastatic potential was independently of the EMT and the EMT was not associated with the metastasis of the DU145 cell line. The mechanism for the decrease in the metastasis with the knockdown of PSCA had not been revealed. Additional studies are warranted.

## Conclusions

In conclusion, to our knowledge, this study is the first to provide evidence that knockdown of PSCA induced EMT and reduced metastatic potentials of the DU145 cells. It suggested that PSCA played an important role in prostatic carcinogenesis and progression.

## Methods

### Cell lines and cell culture

The human prostate epithelial cell line RWPE-1, human PCa cell line PC3, PC3 M, DU145, and 293T were purchased from Type Culture Collection of the Chinese Academy of Sciences (Shanghai, China). The RWPE-1 cells were cultured in Keratinocyte Serum Free Medium (Gbico, Grand Island, NY, USA) and 1 % penicillin and streptomycin combination (KeyGEN BioTECH, Nanjing, China). The DU145 cells and The PC3M cells were maintained in RPMI1640 (Gbico) supplemented with 10 % fetal bovine serum (Gbico) and 1 % penicillin and streptomycin combination. The PC3 cells were cultured in Ham’s F-12K (Kaighn’s) Medium (Gbico) with 10 % fetal bovine serum and 1 % penicillin and streptomycin combination. 293T cells were grown in Dulbecco’s Modified Eagle’s Medium (DMEM) (Gibco) supplemented with 10 % fetal bovine serum and 1 % penicillin and streptomycin combination. All the cells grew in standard cell culture conditions (5 % CO_2_, 95 % humidity) at 37 °C.

### Antibodies and immunoblotting

Western blot analysis was conducted as previously published by our laboratory [30]. Briefly, DU145 cells were collected and washed three times with phosphate-buffered saline (PBS). Next each well was lysed in RIPA buffer. Protein concentrations were determined using the BCA protein assay regents (#23225, Thermo Pierce, Rockford, IL, USA), Equal amount of protein aliquots (40–100 ug) were separated on a 10 % SDS polyacrylamide gel and transferred to a polyvinylidene difluoride (PVDF) membrane (Millipore, Billerica, MA, USA). The membranes were blocked in 5 % non-fat dry milk in PBS-T for 2 hours at room temperature and incubated with primary antibodies overnight at 4 °C. The primary antibodies used included antibodies against PSCA (ab56338, 1:1000 dilution, Abcam, PRC-Cambrige, UK), E-cadherin (cat.#1702-1, 1:1000 dilution, Epitomics, Burlingame, CA, USA), β-catenin (sc-7963, 1:500 dilution, Santa Cruz Biotechnology, Santa Cruz, CA, USA), Vimentin (sc-5565, 1:500 dilution, Santa Cruz Biotechnology, Santa Cruz, CA, USA), Fibronectin (sc-9068, 1:500 dilution, Santa Cruz Biotechnology, Santa Cruz, CA, USA), GAPDH [6C5] (ab8245, 1:3000 dilution, Abcam). The secondary antibodies were anti-mouse and anti-rabbit IgG conjugated with HRP (ab136815, ab136817, 1:3000 dilution, Abcam), The bounded secondary antibodies were reacted to the ECL detection reagents (#1856135, Thermo) and exposed to X-ray films (#47410, Fujifilm, Tokyo, Japan).

### Immunofluorescent staining

The cells were cultured in a 24-well plate and left to grow until reaching 80 % confluence. The cells were then washed three times with PBS for 5 min. Next they were fixed with 4 % paraformaldehyde for 20 min at room temperature, followed by permeabilization with 0.5 % Triton (lot#84704, Invitrogen, Carlsbad, CA, USA). Nonspecific binding sites were saturated by incubation with 5 % BSA for 30 min at 37 °C in the cell incubator. The cells were incubated with PSCA, Ecadherin, or Vimentin antibody overnight and washed with PBS three times for 5 min. They were then incubated with secondary antibody (Alexa Fluor^®^ 594 goat anti-rabbit IgG, Life Technologies, USA)for 1 h and washed three times with PBS for 5 min. A drop of DAPI was added. After the DAPI was evenly distributed, the 24-well plate was visualized using an inverted microscope (ix73, Olympus, Tokyo, Japan).

### Generation of the stable knockdown of PSCA cell line DU145

Four target sequences of the small hairpin RNA for human PSCA were designed (Table 1). The sequences were annealed and cloned into the psi-LVRH1GP vector. After successfully construction of the correct recombined plasmids, the target sequence of the small hairpin RNA was screened for the best effect on the knockdown of PSCA. The best target sequence was found to be CCTGACCGTCATCAGCAAAGG(shRNA#1). The resulting vectors were then co-transfected along with the Gag/Pol, Rev, VSV-G vectors into the 293T cells. The retroviruses were added into DU145 cells for the infection. The positive clones were selected in puromycin (5 ug/ml). The stable PSCA transfectants were isolated after 2 weeks of selection.

### RT-PCR

Total RNA from DU145 cells was isolated with TRIzol (Invitrogen). The mRNA quality was evaluated by the OD260/280 ratio and only samples with a ratio of 1.8–2.0 were used. The total RNA (1 µg) was reversely transcribed to the cDNA using PrimeScript^®^ RT reagent Kit (AK2802, Takara, Shiga, Japan) according to the manufacturer’s standard operating procedure. The PCR (cat.#KT201, TIANGEN, Beijing, China) protocol consisted of cycling at 94 °C for 3 min, followed by 30 cycles of 94 °C for 30 s, 55 °C for 30 s, and 72 °C for 60 s, and a final extension at 72 °C for 5 min. The primers used were shown in Table 2. GAPDH was chosen as the internal control. The PCR products were electrophoresed on a 1 % agarose gel and the bands were visualized with ethidium bromide. Results were normalized with those for GAPDH.

**Table 1:** Designed four target sequence for the small hairpin RNA for the PSCA

Clone name	Location	PSCA target sequence
PSCA shRNA#1	229	CCTGACCGTCATCAGCAAAGG
PSCA shRNA#2	294	GCAAGAAGAACATCACGTGCT
PSCA shRNA#3	528	GAGGCACATCCTAACGCAAGT
PSCA shRNA#4	841	GGCTGAGATGAAGTGGACTGA
Scramble		GCTTCGCGCCGTAGTCTTA

**Table 2:** Primers used for detection of mRNA Expression by RT-PCR

Gene	Sequences	
	Forward	Reverse
Human experiments		
PSCA	GCTGCTTGCCCTGTTGATGG	ACATGGTCAGACTTGCGTTAGGA
GAPDH	CCATCACTGCCACCCAGAAGAC	GTGTCGCTGTTGAAGTCAGAGGAGA
E-cadherin	TCCTCCCAATACATCTCCCTTCA	TCTCCGCCTCCTTCTTCATCATA
Vimentin	TTCGCCAACTACATCGACAAGG	TTCAAGGTCAAGACGTGCCAG

### Real-time quantitative PCR

Using the reversely transcribed cDNA in the prior experiment, real-time quantitative RT-PCR was performed using the SuperReal PreMix Plus (SYBR Green) (cat.#FP205, TIANGEN). Selected sequences of the sense and antisense primers were used for the amplification (Table 3). The PCR thermal cycling conditions consisted of an initial denaturation at 95 °C for 15 min, followed by 40 cycles of denaturation at 95 °C for 10 s, annealing at 58 °C for 20 s, and a 30 s extension at 72 °C. All samples were processed in triplicates and all values were normalized for the GAPDH expression levels. Relative quantification of mRNA levels was determined by ΔΔCt method.

**Table 3:** Primers used for detection of mRNA Expression by qRT-PCR

Gene	Sequences	
	Forward	Reverse
Human experiments		
PSCA	CCTAACGCAAGTCTGACCATGTATG	TGCAGGCGGATCTGTGTCAATA
GAPDH	CGCTGAGTACGTCGTGGAGTC	GCTGATGATCTTGAGGCTGTTGTC
E-cadherin	TCACGCTGTGTCATCCAACGG	TCACGCTGTGTCATCCAACGG
β-catenin	CAGGAAGGGATGGAAGGTCTC	TACCACCCACTTGGCAGACC
Vimentin	ATCCAAGTTTGCTGACCTCTCTGA	GACTGCACCTGTCTCCGGTACTC
Fibronectin	GAGACACCTGGAGCAAGAAGG	GTGAGGCTGCGGTTGGTAAAC
Snail	CTTCTCCTCTACTTCAGTCTCTTCC	TGAGGTATTCCTTGTTGCAGTATTT
Slug	AACAGAGCATTTGCAGACAGGTC	GCTACACAGCAGCCAGATTCC
Twist	CACCATCCTCACACCTCTGCATT	GCTGATTGGCACGACCTCTTGA

### Transwell migration assay

Cell migration was measured using sterile 6.5 mm Transwell^®^ with 8.0 µm pore polycarbonate membrane insert (#3422, Corning, Cambridge, MA, USA). 1 × 10^5^ DU145, DU145 scramble, and DU145 shRNA#1 cells were loaded onto the top of a 24-well migration chamber in 100ul serum-free RPMI 1640 medium. The lower chamber was filled with 0.75 ml of the RPMI 1640 containing 10 % FBS. After 24 h incubation, cells that had migrated into the lower surface of the filter were fixed with 4 % paraformaldehyde and were stained with 0.1 % crystal violet solution. Cells were counted in nine random fields per insert.

### Transwell invasion assay

The same sterile Transwell membrane insert was used to perform the invasion assay. Before commencing the experiment, the BD matrigel (#356234, BD Biosciences, Franklin Lakes, NJ, USA) was placed in a −4 °C refrigerator for 24 h. The BD matrigel would turn into liquid. It was diluted to 1:9 ratios with RPMI 1640. The bottom of the Transwell upper chamber was filled with 50 ul of the diluent. The Transwell insert was incubated for 3 h until the matrigel clotted. The similar steps were performed as the migration assay: 1 × 10^5^ DU145 cells were loaded into the upper chamber of a 24-well matrigel invasion assay plate, the lower chamber was filled with 0.75 ml of RPMI 1640 medium containing 10 % FBS. After 24 h of incubation, cells that had migrated to the lower surface of the filter were fixed with 4 % paraformaldehyde and stained with 0.1 % crystal violet solution. Cell count was performed in nine random fields per insert.

#### In vivo study

Fifteen 6-week-old male BALB/c nude mice were supplied by the Experimental Animal Center of Guangdong province, China. All experimental animal protocols were approved by the Animal Care and Use Ethics Committee. The animals were maintained in a special pathogen free facility. All the mice were randomly divided into three groups of five mice each. DU145 cells were collected, washed, and resuspended in serum-free medium at a concentration of 5 × 10^7^ cells/ml. Aliquots of 100 ul cells from each group were subcutaneously injected into the right side of axillary region of each animal. The animals were followed for 4 weeks, the mice were sacrificed, and the tumors were dissected and put them into liquid nitrogen container for Quantitative RT-PCR and Western blot.

### Statistical analysis

All of the experiments were performed in triplicates. Data were presented as the mean ± SD. The differences between groups were analyzed using one-way analysis of variance. All the statistical analyses were performed using the SPSS 18.0 software. P < 0.05 were considered to be statistically significant.
